# A morphological and functional basis for maximum prey size in piscivorous fishes

**DOI:** 10.1371/journal.pone.0184679

**Published:** 2017-09-08

**Authors:** Michalis Mihalitsis, David R. Bellwood

**Affiliations:** 1 College of Science and Engineering, James Cook University, Townsville, QLD, Australia; 2 Australian Research Council Centre of Excellence for Coral Reef Studies, James Cook University, Townsville, QLD, Australia; Department of Agriculture and Water Resources, AUSTRALIA

## Abstract

Fish predation is important in shaping populations and community structure in aquatic systems. These predator-prey interactions can be influenced by environmental, behavioural and morphological factors. Morphological constraints influence the feeding performance of species, and interspecific differences can thus affect patterns of resource use. For piscivorous fishes that swallow prey whole, feeding performance has traditionally been linked to three key morphological constraints: oral gape, pharyngeal gape, and the cleithral gape. However, other constraints may be important. We therefore examine 18 potential morphological constraints related to prey capture and processing, on four predatory species (*Cephalopholis urodeta*, *Paracirrhites forsteri*, *Pterois volitans*, *Lates calcarifer*). Aquarium-based experiments were then carried out to determine capture and processing behaviour and maximum prey size in two focal species, *C*. *urodeta* and *P*. *forsteri*. All four species showed a progressive decrease in gape measurements from anterior to posterior with oral gape ≥ buccal ≥ pharyngeal ≥ pectoral girdle ≥ esophagus ≥ stomach. *C*. *urodeta* was able to process prey with a maximum depth of 27% of the predators’ standard length; for *P*. *forsteri* it was 20%. *C*. *urodeta* captured prey head-first in 79% of successful strikes. In *P*. *forsteri* head-first was 16.6%, mid-body 44.4%, and tail-first 38.8%. Regardless of capture mode, prey were almost always swallowed head first and horizontally in both focal species. Most internal measurements appeared too small for prey to pass through. This may reflect the compressibility of prey, i.e. their ability to be dorsoventrally compressed during swallowing movements. Despite examining all known potential morphological constraints on prey size, horizontal maxillary oral gape in a mechanically stretched position appears to be the main morphological variable that is likely to affect maximum prey size and resource use by these predatory species.

## Introduction

Coral reefs are exceptionally diverse ecosystems, being home to a plethora of fish species that exhibit an unparalleled diversity of feeding strategies [1]. One of the key questions in coral reef ecology is what determines the nature of prey for piscivorous fishes. One critical, yet poorly understood aspect, is their maximal performance capabilities, i.e. what constrains maximum prey size [2]. The functional performance capacity of a species, i.e. the ability of a species to carry out a specific behaviour or task, is frequently linked with a species’ morphology [2]. Performance capacity affects the ecology of a species by constraining the range of environmental resources it is able to exploit, which in turn influences individual fitness through reproductive output and survival. By interacting with environmental factors, performance capacity constrains, and thus shapes, patterns of resource use [2–4].

Predator-prey outcomes can be influenced by both pre-capture (e.g. prey evasive behaviour) and post-capture factors (e.g. predator handling times) [5, 6]. Pre-capture features focus on prey behaviour and morphological anti-predatory mechanisms [5, 7–10], whereas post-capture features involve morphological constraints, like the ability to capture and manipulate prey. Both pre-capture and post-capture features are tightly linked with ecosystem-wide mortality rates [11]. In piscivory, post-capture features have been studied extensively in terms of optimal foraging theory, investigating aspects such as handling time and energy maximization [8, 12, 13]. However, our understanding of morphological constraints on piscivores is largely restricted to generalized morphological measurements such as body size or mouth gape [8, 14–17].

Piscivorous fishes are often considered to be gape-limited [5, 18–20]. This suggests that mouth size is the main factor limiting prey size, thus making it a good indicator of maximum prey size [5, 18, 21]. For example, Christensen [5] and Hambright [18] both suggest that mouth width is an important morphological constraint of maximum ingestible prey size for both capturing and swallowing prey. However, Lawrence [21] suggested that the gape between the cleithral bones (pectoral girdle) may be an important constraint, while Christensen [5] identified a potential esophageal constraint. More recent studies have also identified the pharyngeal gape as a potential constraint in pharyngognathous fishes [22]. Overall, there appears to be three potential constraints limiting performance in piscivorous fishes that swallow their prey whole: oral gape, pharyngeal gape, and cleithral gape. However, these constraints, and their potential influence on performance, have not been critically or quantitatively assessed in a comparative framework.

Another potential problem with studies of the ecology of predatory fishes, is that despite predators being consistently considered `gape limited`, there appears to be no consistent definition or morphological measurement associated with the term `gape`. While some studies use the term `mouth diameter`[23] or `gape`[24–26] to refer to the distance between the tip of the premaxilla and the tip of the mandible (vertical plane), other studies use the term `gape`to refer to the horizontal distance between the coronoid process of the articular bones (horizontal plane) [27]. Some studies have measured `gape`by inserting a cone into the oral cavity of fish and measuring the diameter [19, 28], while other studies specifically use the term `external mouth width`[18] to measure the distance between the outer edges of the opposing maxillae (horizontal plane) on the outside of the predators’ jaws. More recent studies use the terms `gape width`[29, 30], or `relative mouth width`[31]. In some cases the exact morphological basis for the measurement is not specified [14, 28]. Overall, there appear to be 6 different definitions for the term `gape`, including both vertical and horizontal measurements [19, 25, 27, 29, 32, 33]. There is therefore a clear need to: a) define the various gapes with an exact morphological definition, b) quantify the relative sizes of these gapes in a comparative framework, and c) examine the relationship between gape sizes and maximum prey size in piscivorous fishes.

When capturing prey, piscivorous fish often reorient prey post-capture into a head-first position to swallow it [8, 20, 21, 34]. Reimchen [20] showed that prey orientation when swallowing is random for small sized prey, but increased to 90% head-first when prey approached or exceeded the maximum gape of the predator. However, this behaviour has not been considered in the context of potential morphological constraints in prey processing beyond the oral gape. Thus, prey size may not be limited by what can be caught but by what can be swallowed. Indeed, many of the aforementioned studies have focused on maximum prey size in terms of optimal foraging theory and energetic cost-gain relationships and may have underestimated the maximum performance abilities of predatory fishes [12]. To date, we have not established the maximum ingestible prey size of piscivorous fishes in terms of morphological constraints, and we are therefore unable to establish a clear link between morphology and maximal feeding capacity in piscivorous fishes.

Furthermore, few studies have accounted for the ability of tissues to stretch, a variable that can affect the maximum ingestible prey size, not only in fishes [35], but also other vertebrates that swallow their prey whole, such as snakes [36]. Overlooking the ability of predator tissue to be stretched, or the body of prey fish to be compressed, may lead to conflicting results, with fish feeding on prey with a body depth larger than the maximum gape of the predator [14, 19, 20]. Finally, when trying to identify morphological constraints, broad comparative, morphological analyses are of great utility because they permit the identification of key measurements, and their relationships with other features, offering a deeper understanding of the nature of constraints and the relative importance of potential limiting gapes [30, 37].

Despite the ecological significance of piscivory we currently lack: 1) a clear definition of the important gapes that are involved in prey capture and processing; 2) the nature and extent of morphological constraints on prey size; and 3) data on the relative extent of tissue stretchability or compressibility in fishes. To address these knowledge gaps, the aim of this study was to evaluate the link between the morphology and performance of piscivorous fish with regards to their maximal abilities to capture and swallow prey whole. Specifically, this study explored the trophic morphology of four predatory fish species to identify potential prey size-limiting gapes and to predict maximum ingestible prey size. We then determined the maximum size of prey that can be successfully captured and ingested in two of the four study species in aquarium-based experiments. By addressing both morphological and performance constraints, our study represents a key step in understanding the nature of morphological constraints in piscivory, and in establishing a functional basis of maximum prey size in piscivorous fishes.

## Materials and methods

This study was divided in two sections. Firstly, we quantified 18 morphological structures (gapes) that may limit prey size associated with prey capture and processing by predatory fishes (*Predicted prey size maxima*). Secondly, we used aquarium-based maximal performance experiments to test whether gape measurements can predict the maximal prey sizes of predatory fishes (*Experimental prey size maxima*).

### Ethics statement

This study was conducted in accordance with the animal ethics guidelines of James Cook University, Townsville, including authorization to maintain in captivity, film, and feed study organisms under the James Cook University Animal Welfare & Ethics Committee animal ethics approvals A2298 and A2181. In order to maintain minimal discomfort, and optimal conditions for both predator and prey organisms, individuals were routinely monitored, fed and housing tanks cleaned, in accordance with the authorized ethical procedures. Data are available in S3, S4, S5 and S6 Tables.

### Predicted prey size maxima

**Fig 1:**
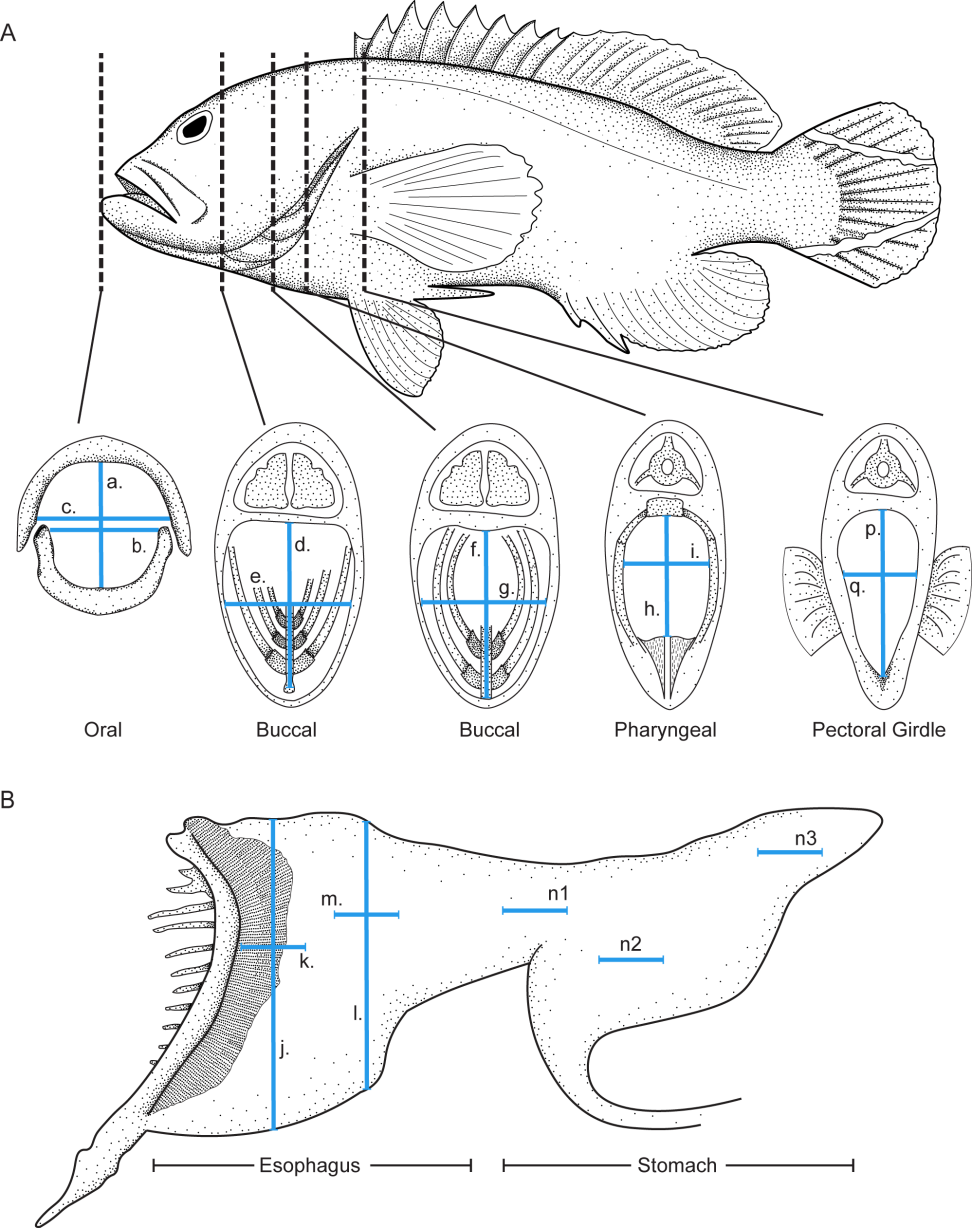
Morphological gape measurements. A) Gape measurements of the oral, buccal, pharyngeal, and pectoral girdle area. B) Gape measurements of the esophagus and stomach. For a detailed description of the measurements, please see Table 1. Please note k, m, and n are internal horizontal measurements.

Exploratory dissections were initially carried out on 5 barramundi (*Lates calcarifer*) (f. Latidae), a catadromous species from the Indo-Pacific region, in order to establish a concise methodology, and to identify a full range of potential gapes that may limit maximum prey size. The average standard length (SL) of these exploratory individuals was 130mm ± 5 standard error (mean ± SE). Individuals were purchased from GFB Fisheries in Kelso, Queensland, Australia. Individuals of this species (n = 3) were also used as a study species in this section as a readily-available analogue of the Indo-Pacific reef barramundi or sand bass (*Psammoperca waigiensis*) (f. Latidae). We also examined *Pterois volitans* (f. Scorpaenidae) (n = 3), a coral reef ambush predator from the Indo-Pacific which has also invaded Caribbean marine ecosystems [38–40]. The two other focal species were *Cephalopholis urodeta* (f. Serranidae), an Indo-Pacific coral reef ambush predator that forages by hiding undercover on the reef, and *Paracirrhites forsteri* (f. Cirrhitidae) an Indo-Pacific coral reef ambush predator that forages by resting on projections on the reef [41]. All coral reef specimens were from the Great Barrier Reef (GBR) and were sourced from commercial aquarium suppliers. Fish were euthanized using clove oil and an ice water slurry. All measurements were taken within one hour of fish euthanasia. External body measurements were taken first: SL, total length (TL), maximum depth (MD), maximum width (MW), and mass (M). Allometric effects were minimized by using individuals of similar sizes (average SL for *C*. *urodeta* individuals was 96.3mm ± 5.75 (mean ± SE), *P*. *forsteri* 91.3mm ± 11.05, *P*.*volitans* 121.3mm ± 1.16, and *L*.*calcarifer* 131.3mm ± 4). Internal gape measurements were taken sequentially, starting from the oral gape, continuing inwards towards the buccal cavity, pharyngeal jaws, esophagus, and stomach (see Table 1 and Fig 1). In total, 18 morphological gape measurements were taken on each individual predator. Exploratory dissections revealed difficulties when measuring internal gapes directly with calipers. Also, it was essential not to cut muscles or soft tissue that might influence the ability of structures or tissues to stretch. We therefore developed a methodology for measuring internal gapes using a specially modified set of dissection scissors. Prior to use, the points of the scissors were individually covered with latex to avoid soft tissue tearing. The front end of the scissors was then inserted into the oral cavity of the fish in the desired position and opened. The width of the back end of the scissors, which was still outside the mouth of the fish, was measured using calipers, recording the distance between the midpoint of the two handles. The scissors were then closed and removed from the oral cavity. The distance between the midpoint of the two handles was then recreated, and the front end of the scissors (which was previously inside the fish) measured using a pair of calipers to record the desired internal gape measurement. It was considered essential to stretch structures and tissues to their mechanical maximum, since previous studies have found a functional mismatch between gapes and prey, with predators being able to feed on prey larger than their measured oral gape [14, 19, 20].

**Table 1:** Summary of internal gape measurements taken on each individual predatory fish.

Morphological measurement	Description
(**a**) Vertical oral gape	Vertical distance between anteriormost upper jaw (premaxilla-premaxilla joint) and lower jaw (dentary-dentary joint)
(**b**) Horizontal articular oral gape	Medial distance between the two articular bones, measured at the dorsoposterior margin
(**c**) Horizontal maxillary oral gape	Maximum medial distance between the left and right maxilla-premaxilla complexes, above the dentary articular margins
(**d**) Vertical first buccal distance	Vertical distance between the dorsal buccal cavity roof just beneath the eye, and the midpoint of the basihyal
(**e**) Horizontal first buccal distance	Horizontal distance between anterior margins of the suspensoria (i.e. between the medial faces of the ectopterygoids)
(**f**) Vertical second buccal distance	Dorsoventral distance between the 2^nd^ basibranchial and roof of the buccal cavity
(**g**) Horizontal second buccal distance	Maximum horizontal distance between the 1^st^ gill arch (1^st^ ceratobranchials)
(**h**) Vertical pharyngeal jaw distance	Dorsoventral distance between the upper and lower pharyngeal jaws (3^rd^,4^th^ pharyngobranchials and 5^th^ ceratobranchials)
(**i**) Horizontal 4^th^ gill arch distance	Horizontal distance between the 4^th^ ceratobranchials
(**j**) Anterior esophagus vertical distance	Dorsoventral distance between the inner walls of the anterior region of the esophagus, located posterior to the pharyngeal apparatus
(**k**) Anterior esophagus horizontal distance	Lateral distance between the walls of the anterior region of the esophagus, located posterior to the pharyngeal apparatus
(**l**) Posterior esophagus vertical distance	Dorsoventral distance between the inner walls of the esophagus located anterior to the stomach
(**m**) Posterior esophagus horizontal distance	Lateral distance between the inner walls of the esophagus located anterior to the stomach
(**p**) Vertical distance of pectoral girdle	Distance between the ventral side of the spinal cord at the pectoral girdle, and the lowest point on the medial margin of the pectoral girdle (where the cleithra join)
(**q**) Horizontal distance of pectoral girdle	Maximum distance between the medial faces of the cleithral bones
(**n1**) Anterior stomach	Maximum distance between the inner walls at the anterior end of the stomach
(**n2**) Mid stomach	Maximum distance between the soft tissue walls at the point where the stomach connects to the intestine
(**n3**) Posterior stomach	Maximum distance between the inner walls at the posterior end of the stomach

The order of measurements is shown according to the order they were taken. Each of these measurements was taken for both first resistance, and mechanical maximum, distending tissues until resistance prevented further movement with light pressure. For an illustration of the measurements on a fish, please see Fig 1.

Following measurements `a-i`(see Table 1 and Fig 1), the upper jaw (maxilla and premaxilla) were removed, along with the soft tissue between the mandible (dentary and articular). The basihyal and urohyal complex were then cut, and the hyoid arch removed. These removals allowed better access to the esophageal and stomach measurements. The pectoral girdle (p and q; Fig 1) was measured by removing connective tissue between the pectoral girdle and the pharyngeal apparatus, and the muscles connecting the upper pharyngeal jaws to the neurocranium (*levator externus*, *levator internus*, and *levator posterior)* to reveal the paired cleithra in situ. The stretchability of soft tissues past the pharyngeal apparatus did not appear to be affected by the aforementioned dissections. In order to take stomach measurements, the pectoral girdle was removed. This was done by cutting the connective tissue binding the pharyngeal apparatus and the esophagus to the pectoral girdle. The anterior end of the visceral cavity was then cut along the dorsoventral axis of the body, and the stomach cut from the intestines before the pyloric caeca, to permit internal gapes to be measured.

### Experimental prey size maxima

We experimentally evaluated the maximum prey size that could be successfully captured and ingested in two focal predatory species: *C*. *urodeta* (n = 3), and *P*. *forsteri* (n = 3). Ethics approval for this study limited the number of individuals per species (JCU Ethics approval A2298). All experiments were carried out at James Cook University, Queensland, Australia, in June and July 2016. Predators were held in individual 20L aquaria, to reduce anti-predatory behaviour by prey fish (see Christensen [5]). Biological filtration for all aquaria was provided by a canister filter and a 90L wet/dry trickle filter with coral rubble. Lighting was turned on every morning at 8-9am and turned off in the evening at 6-7pm. Live *Acanthochromis polyacanthus* were used as prey, as they represent a common natural prey species for piscivores on the GBR [42]. Prey fish were kept in a separate outdoor system in large holding tanks, where they were fed daily with commercially available pellet food.

Initially, predators were fed with small pieces of prawn to acclimatize them to the experimental setup. All fish were acclimatized for at least 10 days. Predators were starved for 24 hours before commencing data collection. Before feeding, a Go-Pro camera was set up in front of the aquarium in order to film the feeding event. For each feeding event, a prey fish was given to a predator after being measured (SL and MD = maximum depth, from the highest point on the body to a point midway between the pelvic and anal fin). Prey fish were measured while held in a plastic bag to avoid skin contact, thus preventing potential effects of handling on predator behaviour due to olfactory cues. Feeding events were classified as either successful or unsuccessful. The predator was given 3 strikes to successfully capture and ingest prey in each feeding event. If after 3 strikes the prey was not captured, the event was considered unsuccessful and the prey fish was removed from the tank. A smaller fish was offered after one hour. Following successful feeding events, predators were allowed to digest prey before initiation of the next feeding event. This digestion period lasted 5–6 days and was assessed by looking at the predators’ external appearance (no swelling in gut area), and its behaviour when we approached the tank. Once ready to feed, fish were again starved for 24 hours and another fish was presented. Where possible, a minimum of six feeding events were recorded for each individual predator. Experimental feeding events were carried out within a three-week time period to minimize changes in the predators’ body size (growth).

Maximum ingestible prey size was defined as the mean between: a) the maximum depth of the smallest prey from an unsuccessful feeding event, and b) the maximum depth of the largest prey from a successful event (see S4 Fig). This value was then standardized to the predators’ SL. Maximum prey size was established for each individual, and a mean ± SE calculated for each species.

In addition to establishing maximum ingestible prey size from successful feeding events, the behaviour of predators in capturing and handling prey was also quantified. Capture mode was quantified in terms of the plane (vertical, horizontal) in which prey was captured, and the location on the body of the first attack (head, mid body, tail). Processing mode was quantified in terms of the orientation in which prey were swallowed (vertical, horizontal). These behaviours were only analyzed from successful strikes.

### Data analysis

All internal gape measurements were standardized to individual predator SL and then the mean (mean ± SE) calculated for each measurement in each species. Regression Tree models were used to identify groupings within the morphological measurements for each species, where gape identities were the predictor variable, and their size the response variable. Analyses were carried out using the rpart package v. 4.1–11 [43] in the software R [44]. Recursive partitioning was applied to the models, with subsequent tree pruning, using Complexity Parameter plots to get the optimal tree size according to Lesmeister [45].

Given the results of the behavioural analyses, only horizontal measurements and the vertical oral gape (see Table 1 and Fig 1) were used in the analyses (all morphological measurements are shown in S1 Fig). Measurements were then grouped a priori according to body part in the following groups: oral, buccal, pharyngeal, esophageal, pectoral girdle, and stomach. Regression Tree models were then reanalyzed on these body regions using the same methodology as above.

Capture and processing modes were quantified for all successful strikes. A successful strike was defined as a strike where the predator was able to successfully capture and hold prey in its mouth for more than three seconds. All successful strikes by a given species of predator were compared to an expected null model where each outcome (prey orientation or strike location), had an equal chance of occurring. Capture orientation (vertical/horizontal) was tested with a binomial test, while capture body part (head, mid, tail) was tested with a χ^2^-test.

Compressibility of prey, i.e. the ability of prey to be dorsoventrally compressed was also quantified. In total, the MD of 38 *A*. *polyacanthus* was measured in a natural state (uncompressed), and also when dorsoventrally compressed with a set of calipers. The force with which prey individuals were compressed, was equivalent to that which the gape measurements of predators were stretched (both compressing and stretching endpoints were characterized by a rapidly increasing degree of resistance to moderate force). Equivalent force by which tissues were stretched and compressed, was applied to a spring scale (PESOLA 1000g). The force expressed as mean grams ± SE from 20 replicates (290.5g ± 8.2) was then converted to Newton (N). The force used was found to be approximately 2.9 N. Possible allometry in the compressibility of prey was examined by measuring compressibility on a wide range of body sizes from juveniles to adults. Compressibility values for prey in experiments were taken from the general relationship between compressibility and SL. We then estimated the mean compressibility ± SE (n = 9 for *C*. *urodeta*, and n = 6 for *P*. *forsteri*) for prey sizes approximating the maximum for each of the two focal fish species. This compressibility was then applied to the estimated maximum prey size that each individual could swallow, from which maximum prey size (when prey is compressed) was estimated for each species (mean ± SE) (n = 3 for *C*.*urodeta*, and n = 3 for *P*. *forsteri*).

## Results

### Morphological constraints on maximum prey size

**Fig 2:**
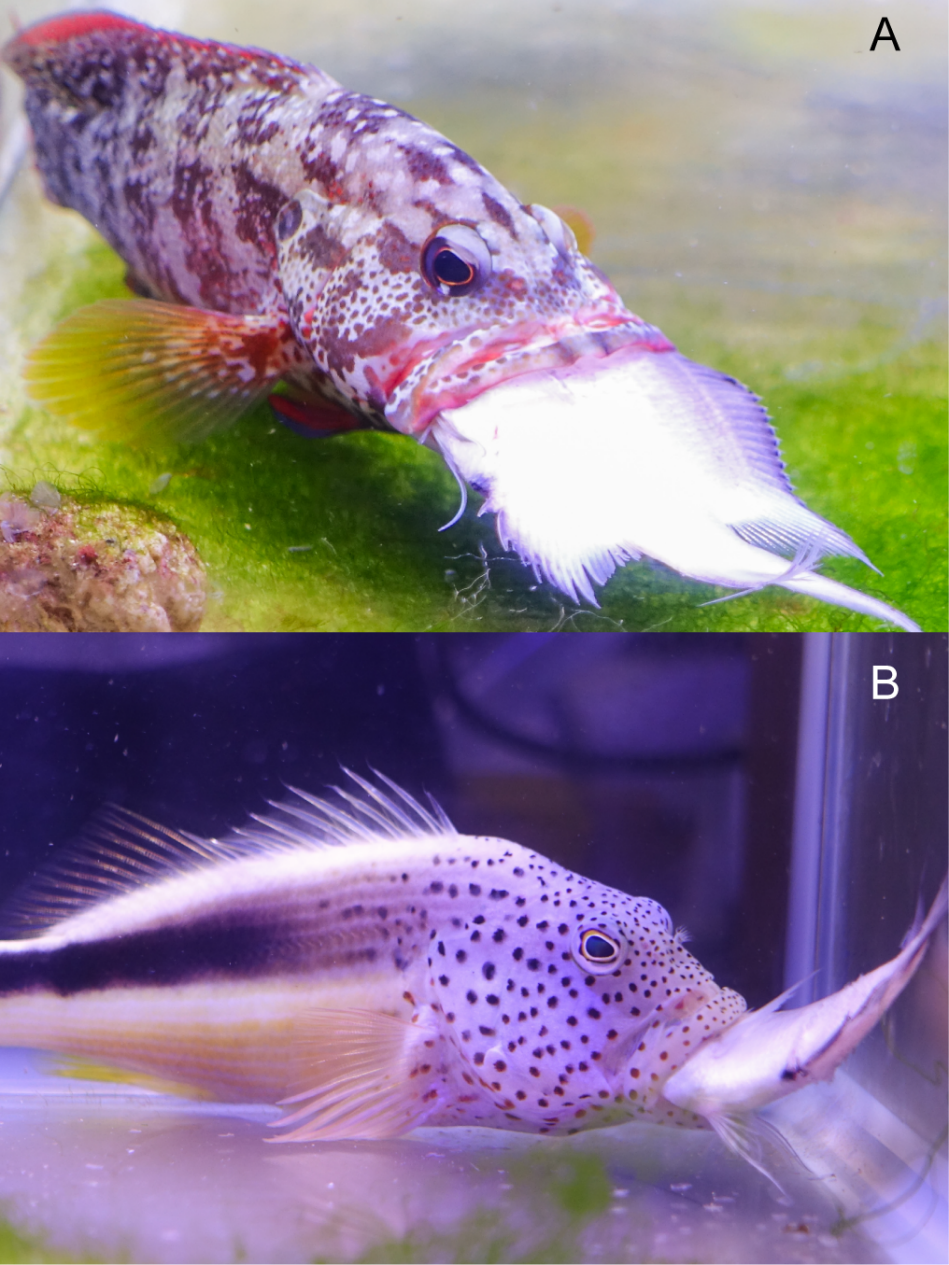
Prey processing by predatory fishes. A) the predator *Cephalopholis urodeta* (SL = 98.4), ingesting a 52.7 mm SL, 27 mm MD *Acanthochromis polyacanthus* in a horizontal, head-first position, and B) a 109 mm SL *Paracirrhites forsteri* that failed to swallow a 45.6 mm SL, 23.1 mm MD, *A*. *polyacanthus*.

**Fig 3:**
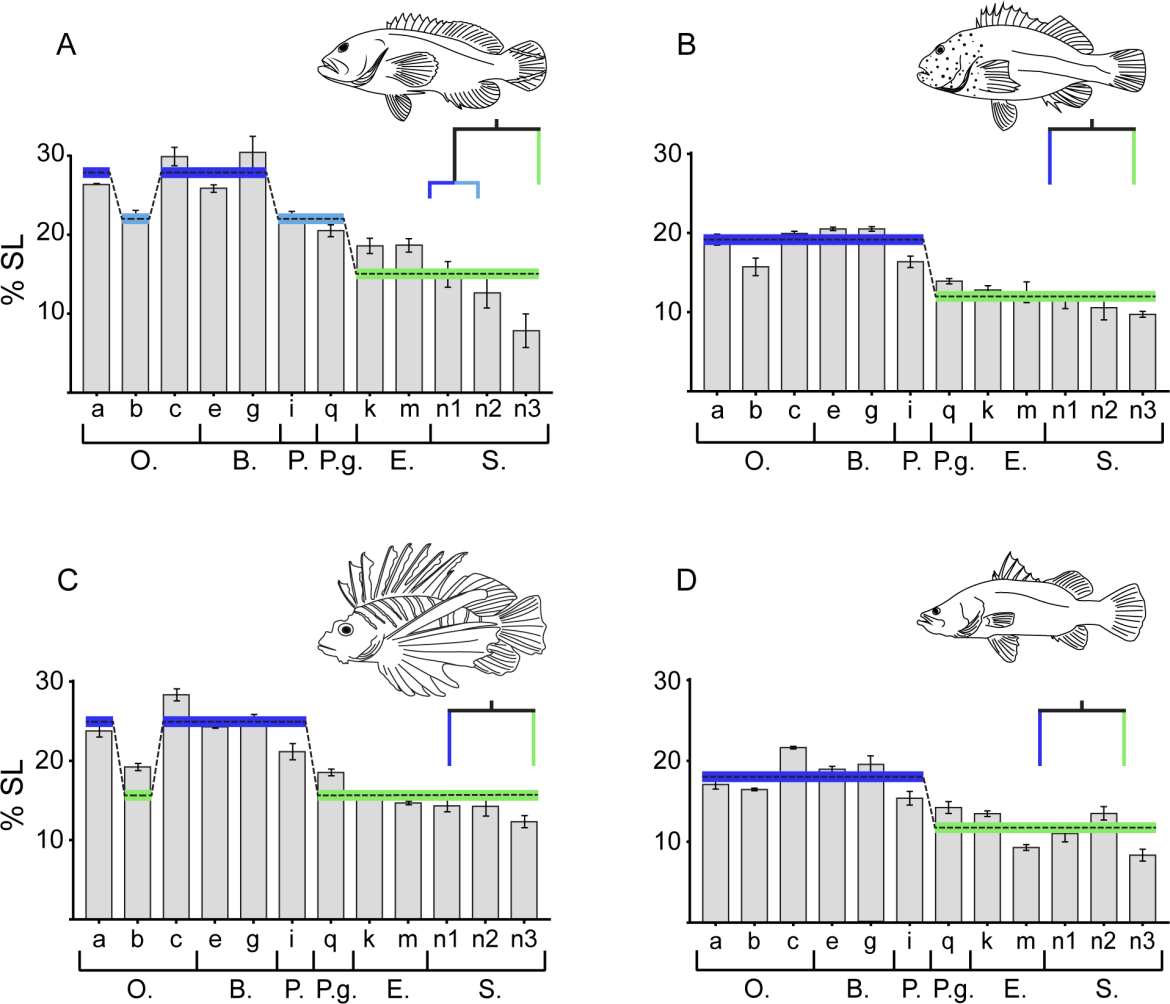
Regression Tree Analysis on key gape measurements. A) *Cephalopholis urodeta*, B) *Paracirrhites forsteri*, C) *Pterois volitans*, and D) *Lates calcarifer*. Measurements (x-axis) are displayed in terms of % of SL (y-axis) (mean ± SE), where shaded horizontal lines represent groupings resulting from the Tree Analysis. Measurements are also grouped into oral (O.), buccal (B.), pharyngeal (P.), pectoral girdle (P.g.), esophagus (E.), and stomach (S.). Colors in graphs represent terminal nodes partitioned by the Regression Tree models.

The predators used in the experiments always swallowed prey in a horizontal position (Fig 2). Therefore, only one vertical measurement was analysed (vertical oral gape, see `a`Table 1 and Fig 1) as it is used in prey capture. However, all horizontal measurements were considered potential constraints during prey ingestion. Altogether, these potentially constraining gape measurements were: a, b, c, e, g, i, q, k, m, n1, n2, n3 (see Fig 1) (for graphs with all measurement see S1 Fig). Despite interspecific differences in the absolute size of gape measurements, the relative size of the gape measurements was remarkably similar among the four species (Fig 3). The Regression Tree model split the data into the following measurement groups in decreasing gape sizes for *C*. *urodeta*: a,c,e,g > b,i,q > k,m,n1,n2,n3 (Fig 3). For *P*. *forsteri*, it was: a,b,c,e,g,i>q,k,m,n1,n2,n3 (Fig 3), *P*.*volitans*: a,c,e,g,i > b,q,k,m,n1,n2,n3 (Fig 3) and for *L*. *calcarifer*: a,b,c,e,g>i,q,k,n2>m,n1,n2 (Fig 3). In essence, in all four species, the Regression Tree model partitioned the various gapes by size into `front gapes`(oral and buccal) and `back gapes`(pharyngeal, pectoral girdle, esophagus, stomach), with the transitioning split (between measurements) being in the rear pharyngeal area (i, q, k, Figs 1 and 3).

When horizontal measurements were grouped *a priori* according to body part, Regression Tree models showed the following splits in decreasing gape sizes for *C*. *urodeta*: oral, buccal, pharyngeal, pectoral girdle > esophagus, stomach (see S2 Fig); for *P*. *forsteri*: oral, buccal, pharyngeal > pectoral girdle, esophagus, stomach (see S2 Fig); for *P*.*volitans*: oral, buccal, pharyngeal > pectoral girdle, esophagus, stomach (see S2 Fig), and for *L*. *calcarifer*: oral, buccal, pharyngeal > pectoral girdle, esophagus, stomach (see S2 Fig). These results suggest that the four species analysed all have a similar relative capacity to capture, swallow and process prey, with oral, and buccal gapes being distinctly larger than pharyngeal and pectoral girdle gapes, which were in turn larger than esophageal or stomach gapes.

### Experimental prey size maxima

**Fig 4:**
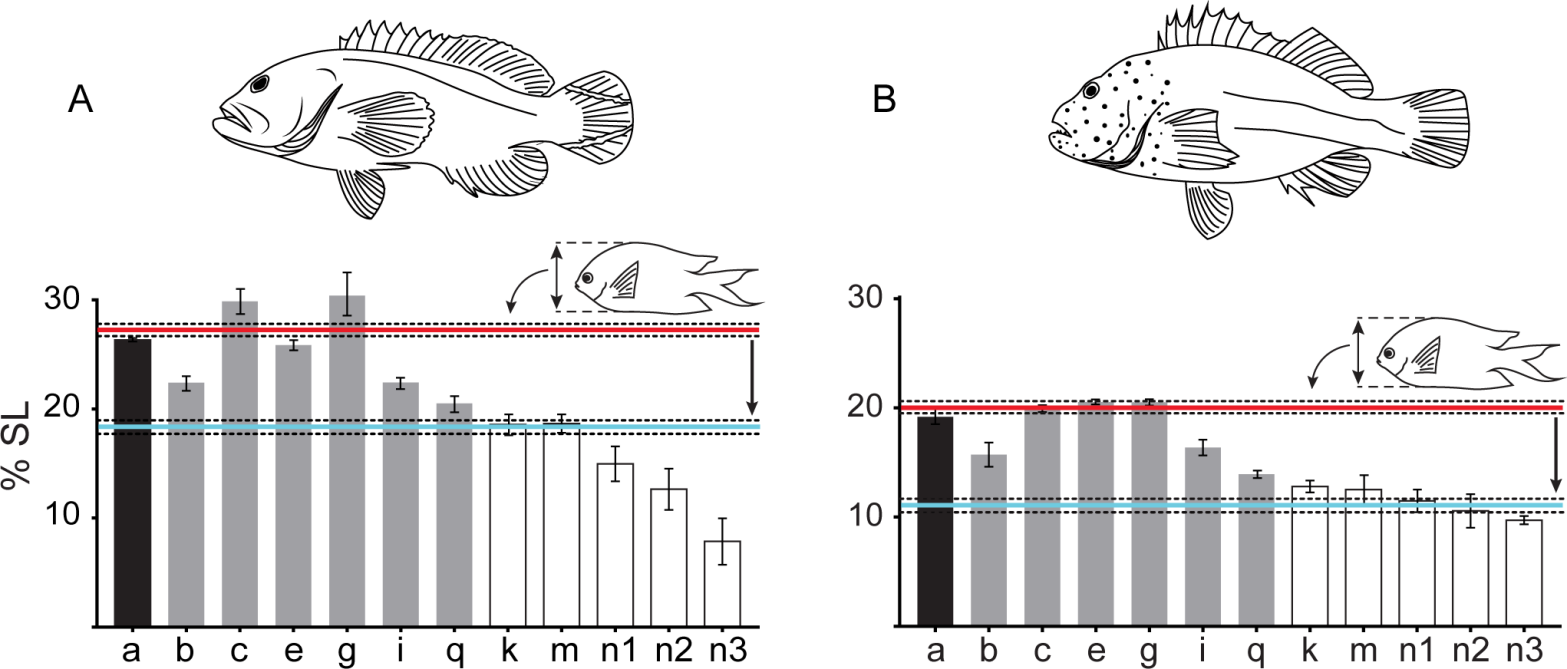
Gape measurements of predators relative to maximum prey size. Gape measurements of A) *Cephalopholis urodeta* (n = 3) and B) *Paracirrhites forsteri* (n = 3) in terms of % SL (mean ± S.E), compared to the mean maximum depth of the largest prey successfully swallowed. Black bars show the vertical oral gape (see `a`Table 1, Fig 1), while grey bars (b, c, e, g, i, q) show hard structure measurements, and white bars (k, m, n1, n2, n3) soft tissue measurements. The red line indicates the mean maximum prey size as a % of predator SL (± S.E dashed lines). The blue line is the mean maximum prey size after accounting for the compressibility of prey (compressed MD).

The mean ratio of maximum prey body size to predator body size in terms of SL was 0.56 ± 0.02 (mean ± SE) for *C*. *urodeta*, and 0.41 ± 0.003 for *P*. *forsteri*. Fish could eat prey approximately half their own length. However, due to the behaviour of predators swallowing prey horizontally, the ratio between mean maximum prey MD and predator SL is probably a more appropriate measure of maximum prey sizes. This ratio was found to be 0.27 ± 0.008 (mean ± S.E.) for *C*. *urodeta*, and 0.20 ± 0.002 for *P*. *forsteri* (Fig 4). This means that *C*. *urodeta* were able to ingest prey with a MD that was 27% of the predators’ SL, whereas *P*. *forsteri* could only ingest prey with a MD that was 20% of predator SL (Fig 4). These results coincide well with oral (c, Fig 4) and buccal (e, g Fig 4) gape measurements. However, most other gape measurements were much smaller than the MD of maximum prey size. While predators should be able to capture (`a`, Fig 4) and engulf prey based on oral and buccal measurements (`c, e, g`, Fig 4), it appears that prey would be unable to pass through the 4^th^ ceratobranchials (`i`, Fig 4) or any further gapes. The fact that fish do swallow these prey suggests that the prey must change in size when swallowed. Prey are likely to be laterally compressed (i.e. dorsoventrally compressed when held in a horizontal position) by the swallowing behaviour of the predators.

**Fig 5:**
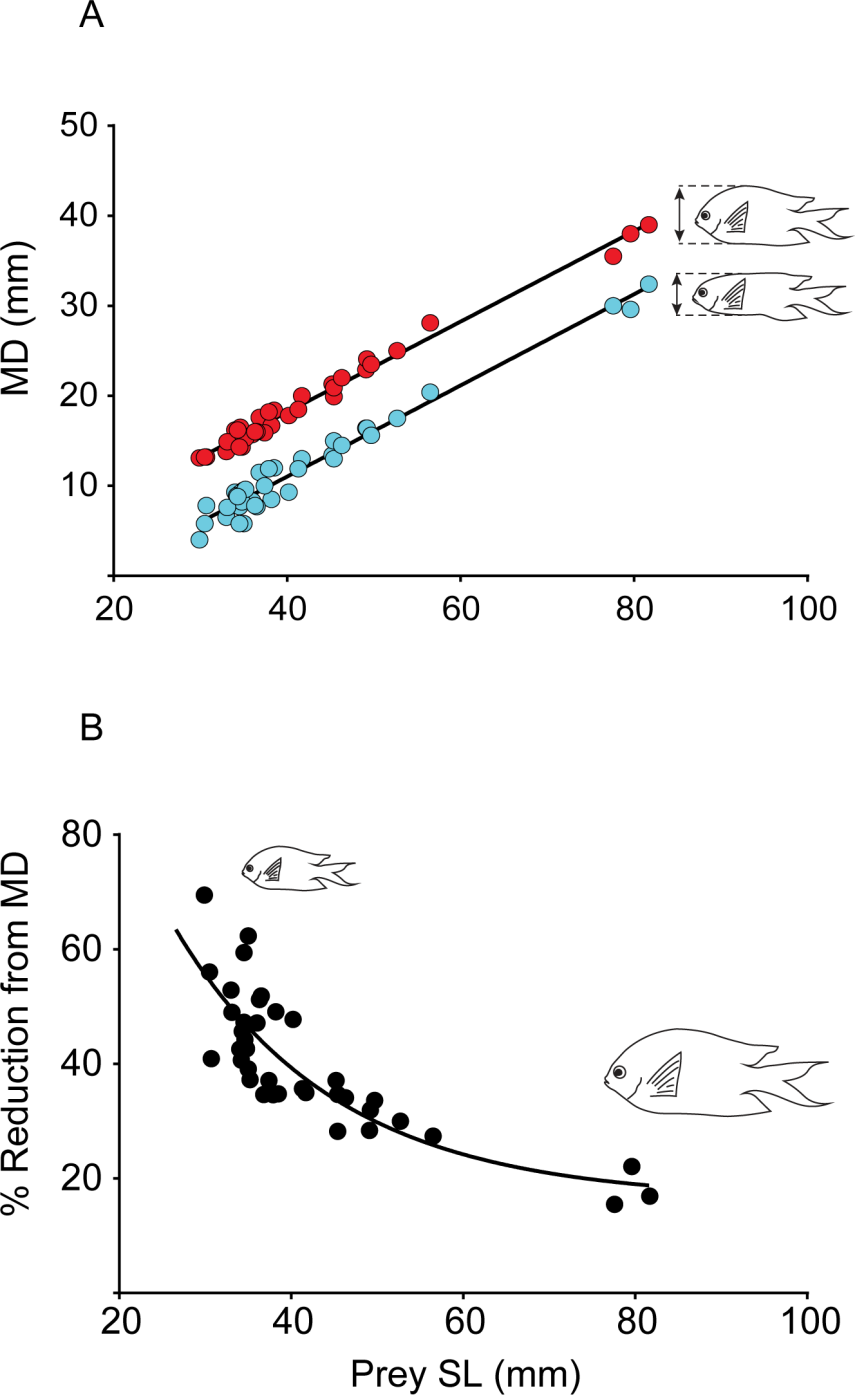
Compressibility of *Acanthochromis polyacanthus*. A) Scatter plot of SL (x-axis) and MD (y-axis) of *A*.*polyacanthus*. Red dots represent natural (non-compressed) MD for individuals, while blue dots represent compressed MD for the same individuals when compressed by a set of calipers with approximately 2.9 N across all sizes. B) Scatter plot of SL (x-axis) and compressibility of individuals, in terms of their ability to be compressed from their natural (non-compressed) MD relative to their size.

The compressibility of *A*. *polyacanthus* appears to vary greatly with body size, with small individuals being proportionally more compressible than larger ones (Fig 5). The mean compressibility of *A*. *polyacanthus* used in experiments for *C*. *urodeta*, was 33.3% ± 1.7 (mean ± SE) (n = 9), whereas for *P*. *forsteri* it was 43.1% ± 4.029 (n = 6). The difference between compressibility calculated for prey of *C*. *urodeta* and *P*. *forsteri*, was because *C*. *urodeta* fed on larger, and thus less compressible individuals than *P*. *forsteri*. When compressibility was accounted for, the mean maximum prey MD to predator SL ratio was 0.18 ± 0.005 (mean ± SE) for *C*. *urodeta*, and 0.11 ± 0.0009 for *P*. *forsteri*. This means that the potential compressed MD of *A*. *polyacanthus* was 18% of *C*. *urodeta* SL, and 11% of *P*. *forsteri* SL (Fig 4).

### Capture and processing behavior

**Fig 6:**
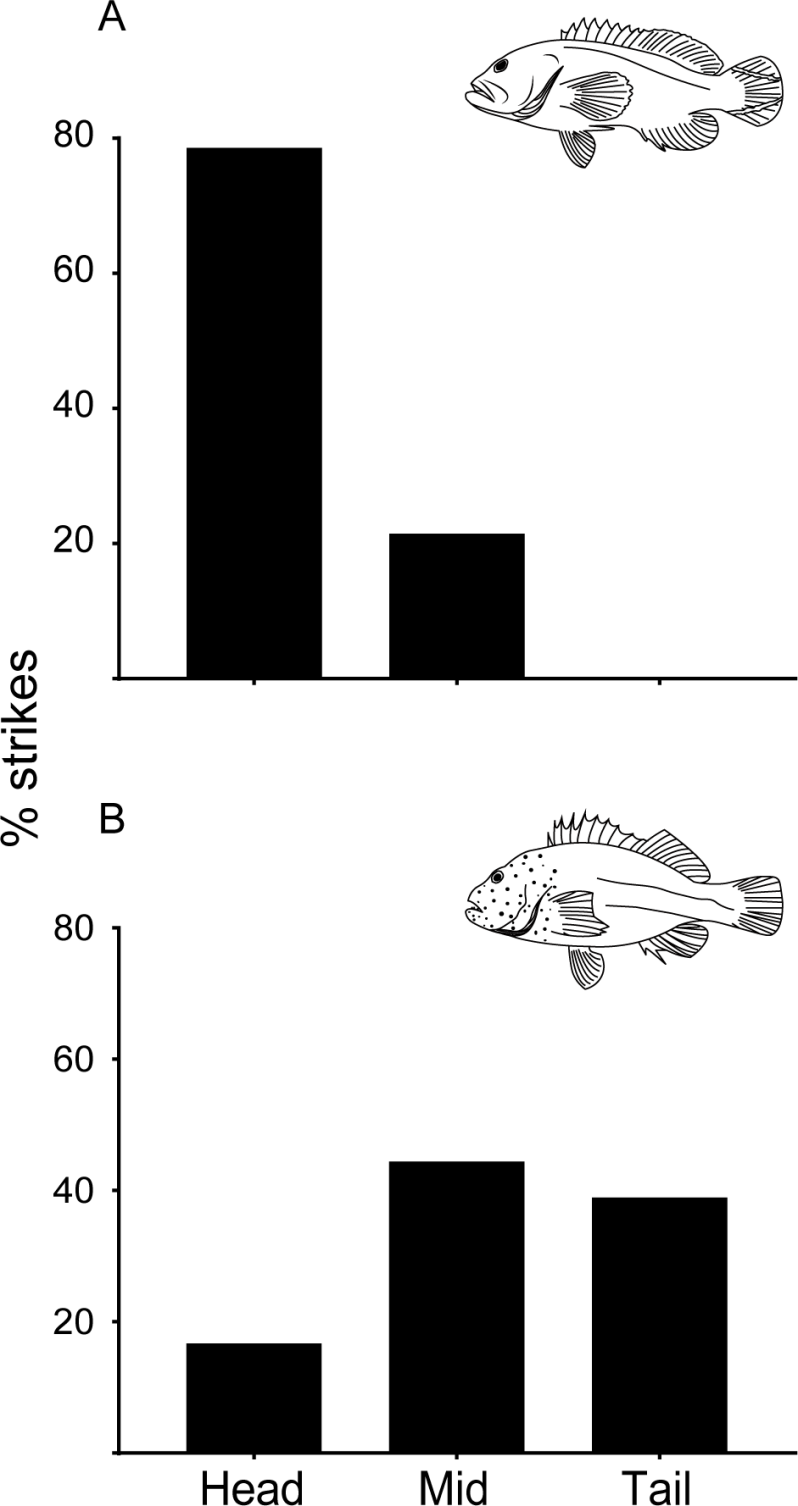
Percentage of successful head-first, mid-body first, or tail-first strikes. A) *Cephalopholis urodeta* (n = 14), and B) *Paracirrhites forsteri* (n = 18).

In total, 32 successful strikes were analyzed of which 14 were for *C*. *urodeta* and 18 for *P*. *forsteri*. *C*. *urodeta* showed no preference for capture mode (p = 0.79) with prey being horizontal in 60% of strikes, and 40% vertical (S2 Table). In contrast, in *P*. *forsteri*, prey capture was always (100%) horizontal (p<0.0001) (S2 Table). *C*. *urodeta* preferably captured prey head-first (79%), followed by mid-body first (21%) (χ^2^ = 13.86, d.f = 2, p = 0.001) (Fig 6 and S1 Table), whereas *P*. *forsteri* showed no preference, and captured prey 16.6% head-first, 44.4% mid-body first, and 38.8% tail-first (χ^2^ = 2.333, d.f = 2, p = 0.3114) (Fig 6 and S1 Table). In general, *C*. *urodeta* grabbed prey, then, if not captured head-first, it reoriented prey into a head-first position by rapid ejection and rebiting. In contrast, the behaviour of *P*. *forsteri* was to attack tail- or mid-body first, then do head-shaking (tearing) movements in a series of jerks and rebites to reorient the prey into a head-first position. Both species swallowed prey horizontally and head-first, except for one feeding event, where a *P*.*forsteri* (SL = 109 mm) swallowed prey (SL = 41.5 mm, MD = 19.5 mm) horizontally but tail-first.

## Discussion

The four piscivorous fish species examined all had a very similar pattern of internal gapes, with oral and buccal gapes being the largest, followed by pharyngeal, pectoral girdle gapes, then esophagus, and stomach gapes. Capture behaviour, however, varied between the two focal species, with *C*. *urodeta* capturing most of its prey head-first, whereas *P*. *forsteri* did not have a preference. Regardless of capture mode, all individuals swallowed prey horizontally and predominantly head-first. Given the ease of measurement and similarity to maximum prey sizes, of the 18 gape measurements considered, the horizontal oral jaw gape (stretched internal distance between the maxillae) was identified as the most reliable and practical gape measurement to estimate maximum prey size in predatory fishes that swallow their prey whole. However, there is a functional mismatch between prey size and gape measurements beyond the oral and buccal regions, especially when prey reach the pharyngeal apparatus and pectoral girdle. This mismatch can be explained if prey compressibility is taken into consideration.

### Morphology and experimental prey size maxima

The 18 internal gape measurements displayed a consistent pattern across the four piscivorous species. The pattern observed herein (Fig 3) is probably similar in other piscivorous fish that swallow their prey whole; however, this needs to be examined by future studies. Our findings suggest that the maximum prey size that a piscivorous fish is able to catch and swallow is well described by the horizontal maxillary oral gape (see Table 1) in a mechanically maximum stretched position (Table 2). The reason for the horizontal oral gape being the best predictor is probably associated with the swallowing behaviour of piscivorous fishes, i.e. turning prey horizontally. The horizontal oral gape of a predator is the gape through which the maximum depth of a prey fish has to pass. It would therefore be advantageous for prey fish to be as laterally compressed as possible. Most reef fish are laterally compressed [46], however, there are some families that have exceptionally deep body depths (e.g. Acanthuridae and Chaetodontidae) suggesting that they have exploited this particular defensive mode to a greater extent than other fishes; possibly because they feed in a manner that makes them particularly vulnerable to predation (cf. [47, 48]). This could suggest a potential evolutionary arms-race between predator and prey in terms of the horizontal oral gape of predators and the maximum depth of prey fish. Morphological prey defence mechanisms include spines, cryptic colouration, and toxicity [49]. The maximum depth of a fish has also been identified as a defence mechanism [50], but it has only been critically evaluated in a few freshwater species (e.g. [7, 18, 19]).

**Table 2:** Summary of predicted and experimental maximum prey size (maximum depth) as a percentage of predator SL.

Species	Predicted	Observed
*Cephalopholis urodeta*	29%	27%
*Paracirrhites forsteri*	20%	20%
*Pterois volitans*	28%	-
*Lates calcarifer*	22%	-

Predicted values are the horizontal maxillary oral gape (see Table 1 and Fig 1) for each species, while experimental (observed) values are the mean MD of the largest prey size that predators were able to successfully capture and ingest under experimental conditions.

Although the present study strongly supports previous suggestions that the horizontal gape of a predator is the best morphological indicator of maximum prey size, it also demonstrated that the horizontal first buccal distance (see Table 1 and Fig 1), might be the narrowest and least flexible of the relevant gapes in the oral-buccal region. This suspensorial gape, and its possible role in constraining prey, would be an interesting avenue for future research as it may limit the ability of fish to fully engulf prey. Other previously suggested morphological constraints that might influence maximum prey size are the cleithra (pectoral girdle) [21], the esophagus [5], and the pharyngeal apparatus [22]. All these features are located in approximately the same region. Our results suggest that during prey swallowing a predator can pass large prey through its mouth and buccal gapes, however, it must somehow reduce prey size when it reaches the pharyngeal/esophagus/pectoral girdle region, as the maximal prey depth can exceed these gapes. One way to circumvent this functional mismatch, is to process prey with the pharyngeal teeth of the pharyngeal apparatus, something described in pharyngognath piscivorous cichlids where prey had been lacerated [51]. Previous studies on the functional abilities of the pharyngeal apparatus of piscivorous fish have focused on pharyngognathy and its influence on pharyngeal gape [22, 52], and pharyngeal jaw morphology [51, 52]. However, other structures may be involved, including the suspensorial system. The results from the present study show a significant drop in gape when transitioning from the buccal to the pharyngeal region (see Fig 3 and S2 Fig). These results suggest that prey may have been laterally compressed (in their dorsoventral plane) by suspensorial muscles in order to get the prey to the pharyngeal region to be further compressed and/or lacerated.

Adduction of the suspensorium can be achieved through contraction of the adductor arcus palatini (AAP) and adductor hyomandibulae (AH) muscles [1, 51]. In combination, the force of the AAP and to a lesser extent the AH, may adduct the suspensorium. We suggest that the resulting force could be used to compress large prey (S4 Fig). Furthermore, previous studies have shown that muscle size, length and angle of attachment to the main tendon (pinnation) have a direct relationship with the force potential of a muscle, and variation within these traits can directly influence the force exerted on a prey item [30, 53]. If suspensorial compression of prey in the buccal area does occur when piscivorous fish swallow prey, the size and fibre angle of the aforementioned muscles, may be of significance.

The present study found that if prey is compressed, prey of the observed maximum size can fit through all internal gapes, except stomach gapes (see n1, n2, n3, Fig 4). Stomach gapes appear to be too small for prey to pass through, even when compressibility is considered. However, during dissections we noticed that if further stretching was applied to stomach tissues over a longer period of time, the tissues could stretch more than initially observed. Stomach gapes are therefore potentially larger than shown in Figs 3 and 4 if the duration of stretching tissues is increased to a few minutes. Also, it must be noted that prey MD does not need to pass through gape n3 (see Fig 4) as it is the posterior end of the stomach, which is where the head of the prey will be located during digestion. If prey position in the stomach, and slow expansion of soft tissue are accounted for, there may be no functional mismatch in any of the aforementioned gapes in the estimation of maximum prey size.

### Feeding behaviour

Piscivores are known to usually capture prey head-first and vertically oriented, then to reorient prey into a horizontal position for swallowing [18, 20, 21]. The results of the present study agree well with the findings of Reimchen [20] who found random swallowing orientation for small prey, but 90% headfirst swallowing when prey approached the horizontal maximum gape of predators. This behaviour has been attributed to avoidance of abrasion, a reduction in escape rates of prey, and a reduction in handling time [20]. The present study demonstrated that for large prey, *P*. *forsteri* attacked tail- or mid-body first, and then reoriented prey into a head-first position, whereas *C*. *urodeta* predominantly aimed for head-first capture. These differences in capturing behaviour reflect the foraging behaviour that these species use in their natural environment, where *C*. *urodeta* hides and feeds within crevices of the reef (see [31, 54]), whereas *P*. *forsteri* rests on the benthos, often on projections on the reef, and attacks prey in the water column (cf. [41]). The one instance where a *P*.*forsteri* swallowed prey tail-first is strange since the ratio of prey MD to predator SL is 0.18, which is close to the maximum prey size ratio of 0.2. This may be associated with the unusual striking behaviour of this species and its preference for attacking tail- and mid-body first. The striking behaviour of predatory reef fishes might therefore be an interesting avenue for future studies.

### Ecological implications

When comparing *C*. *urodeta* and *P*. *forsteri* individuals of similar size, *C*. *urodeta* has larger internal gapes and is thus able to feed on larger prey. These results show how species of similar size may thus show differences in performance. The ecological implications of these results may extend to interspecific differences in patterns of resource use. Previous studies on the maximal performance due to interspecific morphological constraints have shown significant differences in resource use [30]. Such interspecific differences in resource use, and its contribution to prey mortality [11], have the ability to shape population and community dynamics [2, 16, 55]. The results of the present study highlight the importance of performance tests and the maximal capabilities of species. Knowing the maximal feeding potential of species is important, as it sheds light on the potential resource use of species [30], an important aspect of a species fundamental feeding niche.

However, the relationship between the maximal feeding potential, and actual resource use can also shed light on other factors influencing resource use. For example, previous studies investigating maximal feeding performance in fishes, found that in the Caribbean hogfish (*Lachnolaimus maximus*) and some *Halichoeres* labrids, resource use is directly limited by their morphological performance abilities [30, 37]. In contrast, previous studies have shown that piscivorous fishes feed on prey that is smaller than their maximal performance abilities [12]. Although the resource use of piscivorous fishes may be influenced by ecological factors [5] (e.g behaviour, competition), some species, such as the invasive lionfish (*P*. *volitans*) has been found feeding on fish half its size [56, 57]. By establishing a morphological and functional basis for maximum prey size in piscivorous fishes, our study opens new avenues for future studies being able to compare the maximal feeding potential of predatory fishes to their realized feeding habits. Such results can shed light on the influence of factors other than morphology on the feeding habits of predatory fishes, and subsequently permit quantitative comparison of the theoretical feeding niche of species to the realized niches.

The present study found highly congruent patterns of gape sizes among four piscivorous predatory fish species. However, variation in the relative size of the gapes affects the potential performance of two focal predators, with *C*. *urodeta* taking larger prey than *P*. *forsteri*. Internal gapes were consistently smaller than maximum prey sizes, suggesting that piscivores compress prey during ingestion. Overall, of the 18 gapes examined, the horizontal oral jaw gape between the maxillae, was identified as the best indicator of maximum prey size in predatory fishes that swallow their prey whole.

## Supporting information

S1 Fig Gape measurements taken on predators. A) *Cephalopholis urodeta* (n = 3), B) *Paracirrhites forsteri* (n = 3), C) *Pterois volitans* (n = 3), and D) *Lates calcarifer* (n = 3). Measurements (x-axis) are shown in terms of % of SL (y-axis) of individuals (mean ± S.E.). Vertical measurements are shown in gray, whereas horizontal measurements are shown in black. For detailed description of measurements please see Table 1 and Fig 1.
(TIF)

S2 Fig Regression Tree models on gape measurements when grouped according to body part. Measurements are for A) *Cephalopholis urodeta*, B) *Paracirrhites forsteri*, C) *Pterois voitans*, and D) *Lates calcarifer*. Gape measurements (x-axis) are displayed in terms of % of standard length (SL) (y-axis) (mean ± S.E.), where colored horizontal lines, represent groupings resulting from the Regression Tree models.
(TIF)

S3 Fig Theoretical example of how maximum prey size was defined. Green dots represent successful feeding events, while red dots represent unsuccessful feeding events. Maximum prey size was defined as the mean of the maximum depth of the smallest unsuccessful prey, and the largest successful prey item.
(TIF)

S4 Fig Conceptual representation of suspensorial adduction. A) Representation of the bones and muscles capable of suspensorial adduction: nc = neurocranium, su = suspensorium, ih = interhyal, uh = urohyal complex, *aap* = adductor arcus palatini. B) Direction of movement of bones, after muscle contraction.
(TIF)

S1 Table Chi-square test. The body regions that *Cephalopholis urodeta* and *Paracirrhites forsteri* attacked first.
(PDF)

S2 Table Binomial test. Prey orientation when captured by *Cephalopholis urodeta* and *Paracirrhites forsteri*.
(PDF)

S3 Table Raw data. Morphological gape measurements for species used in the analyses.
(PDF)

S4 Table Raw data. Maximum prey size performance experiments.
(PDF)

S5 Table Raw data. Compressibility test on *Acanthochromis polyacanthus*.
(PDF)

S6 Table Raw data. Behaviour of capturing and processing prey by predators.
(PDF)
