# Supplementary figures and images for: A morphological and functional basis for maximum prey size in piscivorous fishes
Source: PLoS One. 2017 Sep 8;12(9):e0184679. doi: 10.1371/journal.pone.0184679 (PMC5590994; doi:10.1371/journal.pone.0184679)

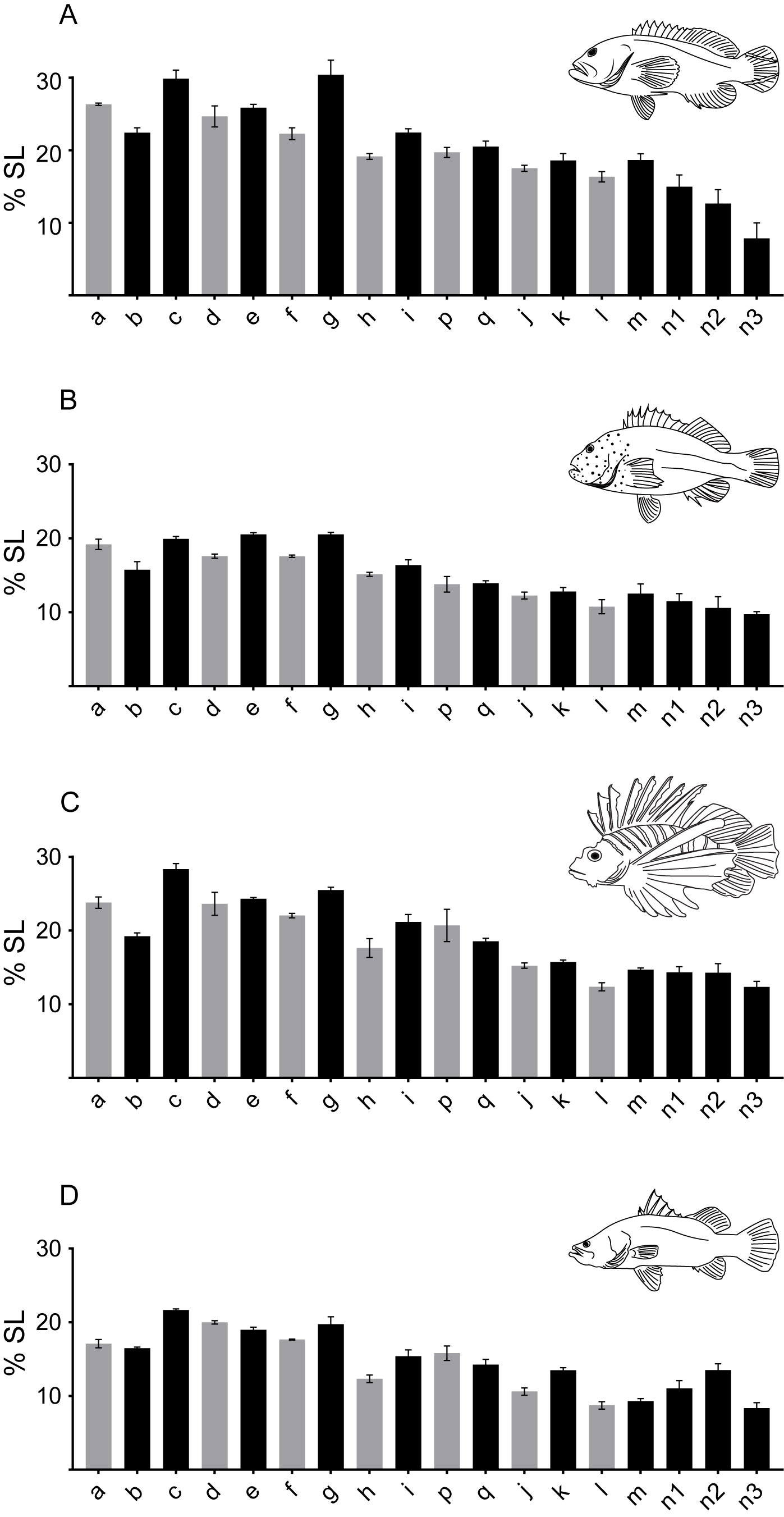

Supplement: S1 Fig — A) Cephalopholis urodeta (n = 3), B) Paracirrhites forsteri (n = 3), C) Pterois volitans (n = 3), and D) Lates calcarifer (n = 3). Measurements (x-axis) are shown in terms of % of SL (y-axis) of individuals (mean ± S.E.). Vertical measurements are shown in gray, whereas horizontal measurements are shown in black. For detailed description of measurements please see Table 1 and Fig 1. (TIF) [file pone.0184679.s001.tif]

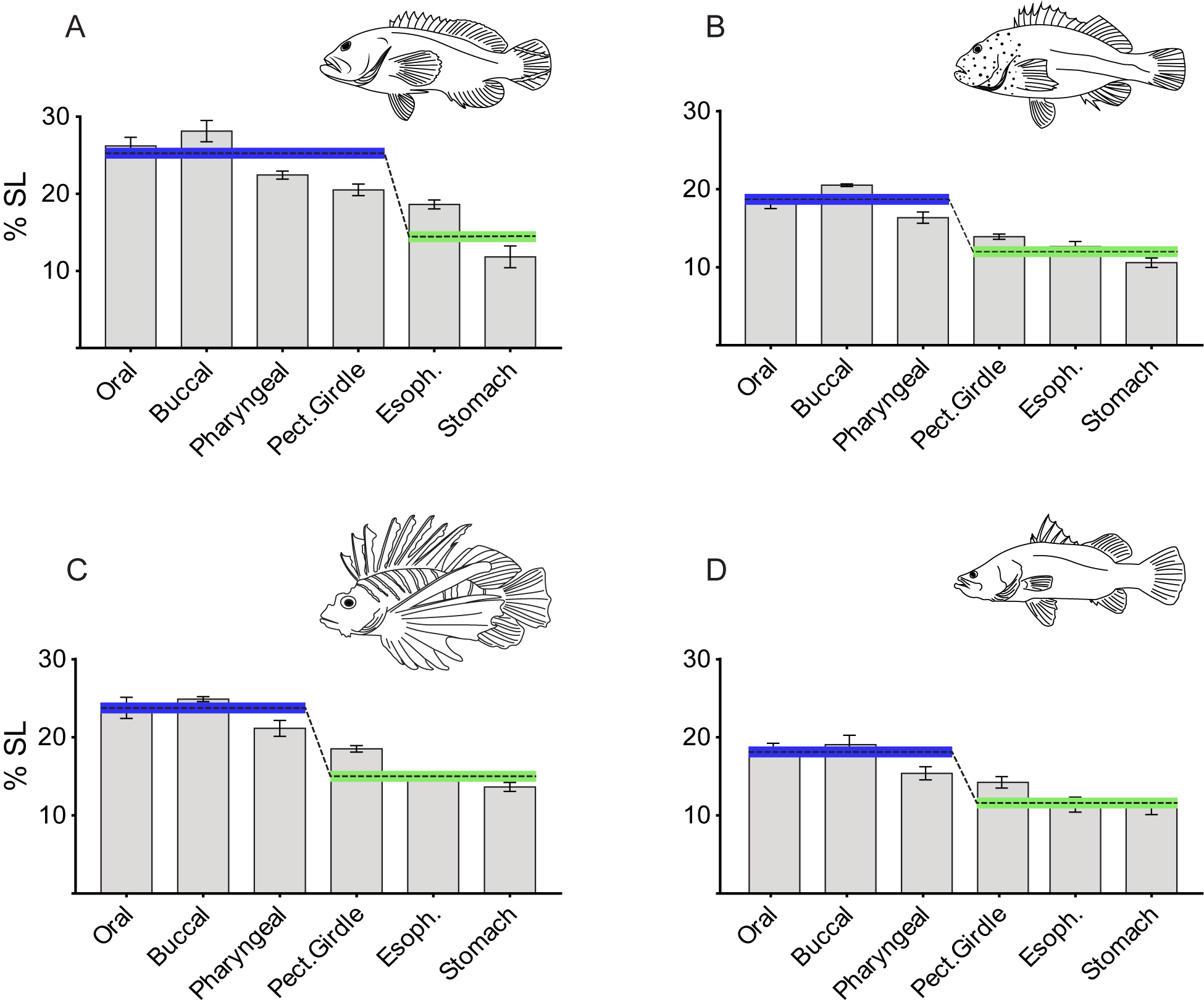

Supplement: S2 Fig — Measurements are for A) Cephalopholis urodeta, B) Paracirrhites forsteri, C) Pterois voitans, and D) Lates calcarifer. Gape measurements (x-axis) are displayed in terms of % of standard length (SL) (y-axis) (mean ± S.E.), where colored horizontal lines, represent groupings resulting from the Regression Tree models. (TIF) [file pone.0184679.s002.tif]

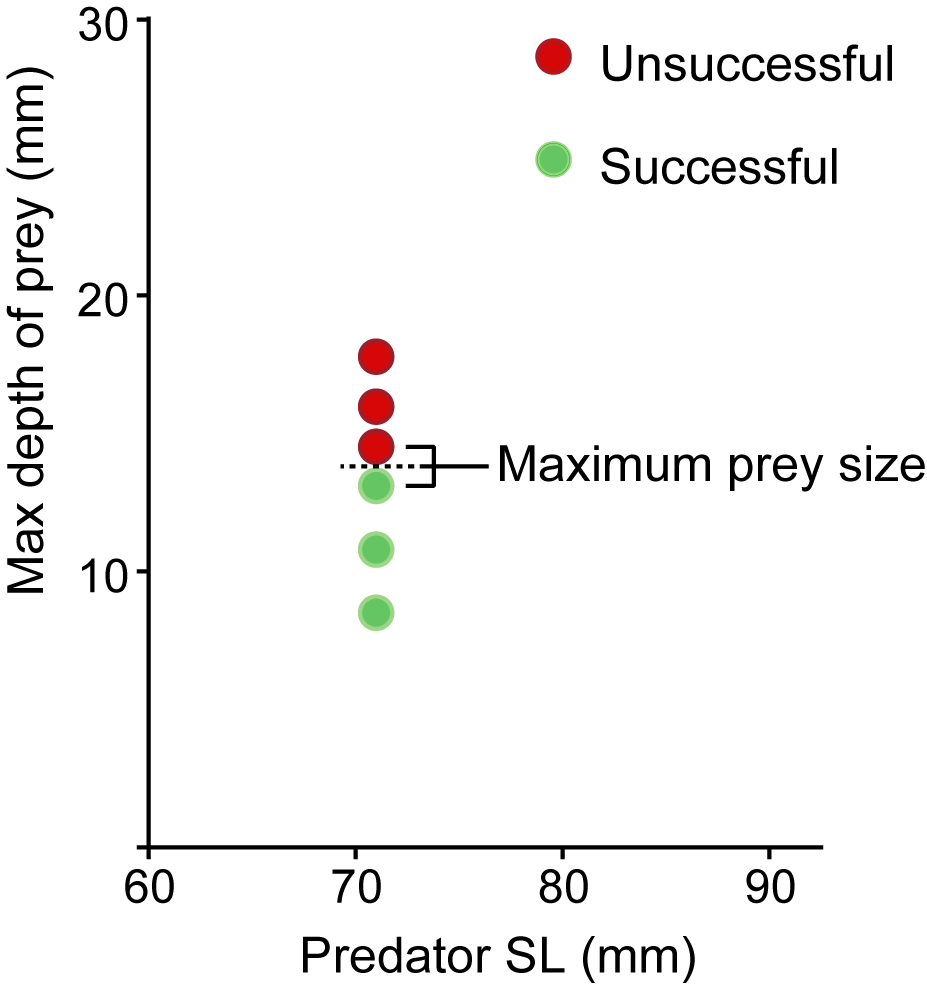

Supplement: S3 Fig — Green dots represent successful feeding events, while red dots represent unsuccessful feeding events. Maximum prey size was defined as the mean of the maximum depth of the smallest unsuccessful prey, and the largest successful prey item. (TIF) [file pone.0184679.s003.tif]

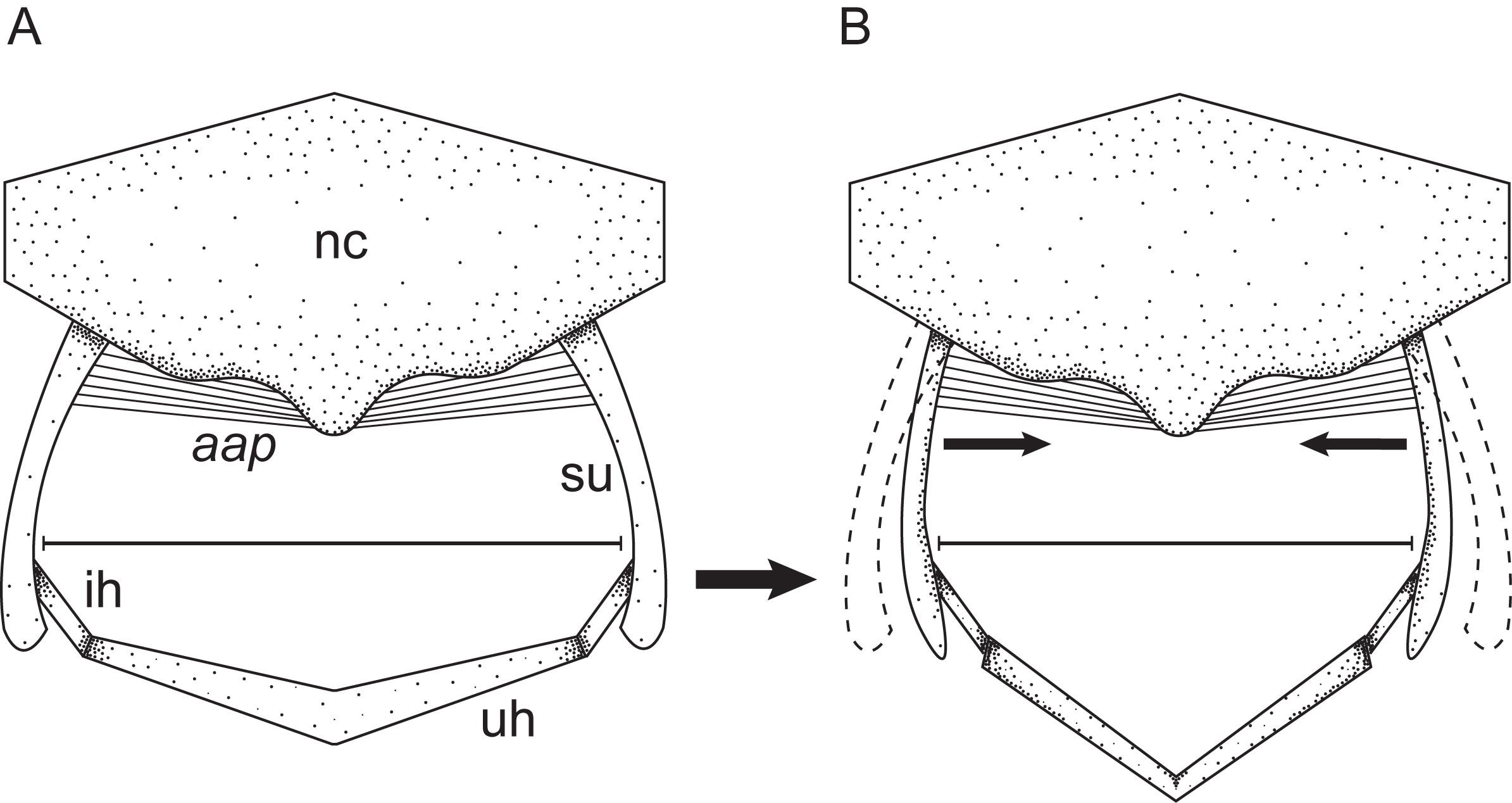

Supplement: S4 Fig — A) Representation of the bones and muscles capable of suspensorial adduction: nc = neurocranium, su = suspensorium, ih = interhyal, uh = urohyal complex, aap = adductor arcus palatini. B) Direction of movement of bones, after muscle contraction. (TIF) [file pone.0184679.s004.tif]
