# Supplementary material for: A morphological and functional basis for maximum prey size in piscivorous fishes
Source: PLoS One. 2017 Sep 8;12(9):e0184679. doi: 10.1371/journal.pone.0184679 (PMC5590994; doi:10.1371/journal.pone.0184679)
Supplement: S1 Table — The body regions that Cephalopholis urodeta and Paracirrhites forsteri attacked first. (PDF) [file pone.0184679.s005.pdf]

S1 Table. Chi-square test. The body regions that *Cephalopholis urodeta* and *Paracirrhites forsteri* attacked first.

Species	Body Part	Observed	Expected	d.f	x^2	p
C.urodeta	Head	11	4.667	2	13.86	0.001
	Mid	3	4.667			
	Tail	0	4.667			
	Sum	14	14			
P.forsteri	Head	3	6	2	2.333	0.3114
	Mid	8	6			
	Tail	7	6			
	Sum	18	18			
