# Supplementary material for: A morphological and functional basis for maximum prey size in piscivorous fishes
Source: PLoS One. 2017 Sep 8;12(9):e0184679. doi: 10.1371/journal.pone.0184679 (PMC5590994; doi:10.1371/journal.pone.0184679)
Supplement: S2 Table — Prey orientation when captured by Cephalopholis urodeta and Paracirrhites forsteri. (PDF) [file pone.0184679.s006.pdf]

S2_Table. Binomial test. Prey orientation when captured by *Cephalopholis urodeta* and *Paracirrhites forsteri*.

Species	Orientation	Observed	Expected	95% C.I	p (2-tailed)
C.urodeta	V	8	7	32.59 to 78.62	0.7905
	H	6	7	21.38 to 67.41	
Sum		14	14		
P.forsteri	V	0	9	0 to 17.59	<0.0001
	H	18	9	82.41 to 100	
Sum		18	18		
