# Supplementary material for: A morphological and functional basis for maximum prey size in piscivorous fishes
Source: PLoS One. 2017 Sep 8;12(9):e0184679. doi: 10.1371/journal.pone.0184679 (PMC5590994; doi:10.1371/journal.pone.0184679)
Supplement: S3 Table — Morphological gape measurements for species used in the analyses. (PDF) [file pone.0184679.s007.pdf]

S3 Table. Raw Data: Morphological gape measurements for species used in the analyses.

Species	SL	a	b	c	d	e	f	g	h	i	p	q	j	k	l	m	n1	n2	n3
Cephalopholis urodeta	105.1	27.9	24.3	29.8	23	28.1	22.4	28.5	20	22.7	19.7	20	17.9	18.6	16.4	20.5	15.5	11.5	7
Cephalopholis urodeta	98.4	25.6	22.7	28.6	26.3	24.7	23.5	33.5	18.2	23.0	19.0	21.1	16.9	20.2	17.5	19.2	17.6	16.2	11.8
Cephalopholis urodeta	85.5	22.6	18	27.5	21.7	22	18.5	25.7	17	19.1	18	18	15.7	15	13.4	14.5	10.5	9	4.2
Paracirrhites forsteri	71	14.6	9.6	14.5	12.6	14.5	12.6	14.2	10.7	10.6	8.8	9.6	8.4	8.4	6.3	7.1	7	7.4	7.4
Paracirrhites forsteri	109	20	18.5	21.1	19.6	22	19.3	22.9	16	18.8	14.3	14.9	12.8	14	12.6	14.4	12.2	8.7	10
Paracirrhites forsteri	94	17.5	15.7	18.8	16	19.7	16.2	19.3	14.7	15.9	14.9	13.7	12.4	12.9	11.1	13.5	12.6	12.5	9
Pterois volitans	119.2	27.4	22.8	32	26.8	28.6	25.6	30	18.1	22.8	27.5	21.2	17.3	18.4	13.6	17.5	16.2	16.8	15.9
Pterois volitans	121.7	28	22.5	35.7	32.5	29.6	27.4	31.9	22.3	26.8	27.6	23.4	18.8	18.9	16.2	17.4	16.5	14.9	13.2
Pterois volitans	123.2	31.2	24.7	35.5	26.7	30.3	27.2	30.9	23.9	27.5	20.1	22.9	19.4	20	15.2	18.6	19.5	20.3	15.8
Lates calcarifer	126.5	22	21	27	25.6	24	22.5	21.93	15.2	17.5	18.1	18.3	12.2	16.2	12.3	12.5	14.5	19.2	11.7
Lates calcarifer	128.2	20.5	20.7	28	25	23.5	22.5	26.19	17.1	20	22.6	16.5	14.5	17.9	10.7	11.1	16.1	16.4	11.4
Lates calcarifer	139.3	24.9	23.2	30.3	28.1	27.3	24.5	28.16	16.2	23.3	21.5	21.4	15.1	19	11.3	13	12.6	17.5	9.6
