# Supplementary material for: A morphological and functional basis for maximum prey size in piscivorous fishes
Source: PLoS One. 2017 Sep 8;12(9):e0184679. doi: 10.1371/journal.pone.0184679 (PMC5590994; doi:10.1371/journal.pone.0184679)
Supplement: S4 Table — Maximum prey size performance experiments. (PDF) [file pone.0184679.s008.pdf]

S4 Table. Raw Data: Maximum prey size performance experiments.

Species	SL	Prey SL	Prey MD	Outcome
Cephalopholis urodeta	105.1	77.8	39	0
		64.7	32	0
		65.3	30.5	0
		47	22.3	1
		49.7	22.9	1
		54.2	26	1
Cephalopholis urodeta	98.4	42.8	23	1
		52.1	24.5	1
		52.7	27	1
		70.5	35.5	0
		65	31	0
		64.7	30	0
Cephalopholis urodeta	85.5	46.8	24	0
		43.3	21	0
		49.5	24.2	0
		35.5	17.2	1
		42.2	21	1
		47	23.5	1
Paracirrhites forsteri	71	30.3	14.9	0
		35	16.3	0
		32.7	15.5	0
		17.2	8.5	1
		29.1	13.1	1
Paracirrhites forsteri	109	48.9	27	0
		47	22.3	0
		45.6	23.1	0
		47.1	22.1	0
		41.5	19.5	1
		43.8	21.4	1
Paracirrhites forsteri	94	51	24.8	0
		46.6	22.3	0
		46.4	22	0
		43	20	0
		40.5	19.2	0
		39.8	18.1	0
		36.9	18.3	1
		36.3	16.7	1
