# Supplementary material for: A morphological and functional basis for maximum prey size in piscivorous fishes
Source: PLoS One. 2017 Sep 8;12(9):e0184679. doi: 10.1371/journal.pone.0184679 (PMC5590994; doi:10.1371/journal.pone.0184679)
Supplement: S5 Table — Compressibility test on Acanthochromis polyacanthus. (PDF) [file pone.0184679.s009.pdf]

S5 Table. Raw Data: Compressibility test on *Acanthochromis polyacanthus*.

SL	MD	MDC
30.7	13.2	7.8
34	16.2	9.3
35	15.6	9.5
36.8	17.6	11.5
38.5	18.4	12
41.7	20	13
45.2	21.3	13.4
45.4	19.9	13
45.4	20.9	15
46.3	22	14.5
49.1	22.9	16.4
49.2	24.1	16.4
49.7	23.5	15.6
52.7	25	17.5
56.5	28.1	20.4
79.6	38	29.6
40.2	17.8	9.3
34.6	16.5	9.2
33	13.8	6.5
35	15.4	5.8
34.2	15	8.9
41.3	18.5	11.9
38.2	16.7	8.5
37.4	15.9	10
36	15.7	8.3
29.9	13.1	4
34.5	14.6	7.7
34.8	14.3	8.2
37.9	18.2	11.9
36.5	16	7.7
30.5	13.2	5.8
33.1	14.9	7.6
35.2	15.3	9.6
34.3	16.2	8.8
34.5	14.3	5.8
36.3	16	7.8
81.7	39	32.4
77.6	35.5	30
