# Supplementary material for: A morphological and functional basis for maximum prey size in piscivorous fishes
Source: PLoS One. 2017 Sep 8;12(9):e0184679. doi: 10.1371/journal.pone.0184679 (PMC5590994; doi:10.1371/journal.pone.0184679)
Supplement: S6 Table — Behaviour of capturing and processing prey by predators. (PDF) [file pone.0184679.s010.pdf]

S6 Table. Raw Data: Behaviour of capturing and processing prey by predators.

Species	SL	Prey SL	Prey MD	Outcome	Suc. Strikes	Orient. Capt.	Capture at	Orient. Proc.	Process starting at
Cephalopholis urodeta	105.1	47	22.3	1	1	V	Head	H	Head
		48.9	27	0	3	V	Head		
						H	Head		
						V	Head		
		49.7	22.9	1	1	V	Head	H	Head
		54.2	26	1	1	H	Mid	H	Head
		64.7	32	0	0				
		65.3	30.5	0	0				
		77.8	39	0	0				
Cephalopholis urodeta	98.4	42.8	23	1	1	H	Mid	H	Head
		52.1	24.5	1	1	H	Head	H	Head
		52.7	27	1	1	H	Mid		
		64.7	30	0	0				
		65	31	0	0				
		70.5	35.5	0	0				
Cephalopholis urodeta	85.5	35.5	17.2	1	1	H	Head	H	Head
		42.2	21	1	1	V	Head	H	Head
		43.4	21	0	1	V	Head	H	Head
		46.8	24	0	0				
		47	23.5	1	1	H	Head	H	Head
		49.5	24.2	0	1	H	Head		
P.forsteri	71	35	16.3	0	1	H	Tail		
		32.7	15.5	0	3	H	Tail		
						H	Mid		
						H	Tail		
		30.3	14.9	0	0				
		29.1	13.1	1	2	H	Tail		
						H	Head	H	Head
		17.2	8.5	1	1		Head		
P.forsteri	109	48.9	27	0	1	H	Mid		
		47.1	22	0	2	H	Tail		
						H	Mid		
		45.6	23.1	0	4	H	Mid		
						H	Tail		
						H	Head		
						H	Head		
		43.8	21.4	1		H	Mid	H	Head

		41.5	19.5	1	1	H	Mid		
						H	Tail	H	Tail
P.forsteri	94	35.7	17.5	0	1	H	Tail		
		36.3	16.7	1	2	H	Mid		
						H	Tail	H	Head
		36.9	18.3	1	1	H	Mid	H	Head
